# High-Dose Intravenous Immunoglobulin in Severe Coronavirus Disease 2019: A Multicenter Retrospective Study in China

**DOI:** 10.3389/fimmu.2021.627844

**Published:** 2021-02-19

**Authors:** Wei Cao, Xiaosheng Liu, Ke Hong, Zhiyong Ma, Yuelun Zhang, Ling Lin, Yang Han, Yong Xiong, Zhengyin Liu, Lianguo Ruan, Taisheng Li

**Affiliations:** ^1^ Department of Infectious Diseases, Peking Union Medical College Hospital, Peking Union Medical College and Chinese Academy of Medical Sciences, Beijing, China; ^2^ Department of Basic Medical Sciences, School of Medicine, Tsinghua University, Beijing, China; ^3^ Tsinghua-Peking Center for Life Sciences, Beijing, China; ^4^ Department of Infectious Diseases, Jin Yin-tan Hospital, Wuhan, China; ^5^ Department of Infectious Diseases, Zhongnan Hospital of Wuhan University, Wuhan, China; ^6^ Medial Research Center, Peking Union Medical College Hospital, Peking Union Medical College and Chinese Academy of Medical Sciences, Beijing, China

**Keywords:** COVID-19, high-dose intravenous immunoglobulin, immunomodulation, 28-day mortality, inflammatory markers

## Abstract

**Background:**

The effective treatment of coronavirus disease 2019 (COVID-19) remains unclear. We reported successful use of high-dose intravenous immunoglobulin (IVIg) in cases of severe COVID-19, but evidence from larger case series is still lacking.

**Methods:**

A multi-center retrospective study was conducted to evaluate the effectiveness of IVIg administered within two weeks of disease onset at a total dose of 2 g/kg body weight, in addition to standard care. The primary endpoint was 28-day mortality. Efficacy of high-dose IVIg was assessed by using the Cox proportional hazards regression model and the Kaplan-Meier curve adjusted by inverse probability of treatment weighting (IPTW) analysis, and IPTW after multiple imputation (MI) analysis.

**Results:**

Overall, 26 patients who received high-dose IVIg with standard therapy and 89 patients who received standard therapy only were enrolled in this study. The IVIg group was associated with a lower 28-day mortality rate and less time to normalization of inflammatory markers including IL-6, IL-10, and ferritin compared with the control. The adjusted HR of 28-day mortality in high-dose IVIg group was 0.24 (95% CI 0.06–0.99, p<0.001) in IPTW model, and 0.27 (95% CI 0.10–0.57, p=0.031) in IPTW-MI model. In subgroup analysis, patients with no comorbidities or treated in the first week of disease were associated with more benefit from high-dose IVIg.

**Conclusions:**

High-dose IVIg administered in severe COVID-19 patients within 14 days of onset was linked to reduced 28-day mortality, more prominent with those having no comorbidities or treated at earlier stage.

## Introduction

The pandemic of coronavirus disease 2019 (COVID-19), caused by a novel coronavirus SARS-CoV-2, has continued to spread since late 2019. Up to date, over 19 million people have been affected globally, and the number is still growing (1). Although the majority of infected individuals had a mild or moderate disease course and recovered without serious sequela, 10–20% of infected patients were classified as severe and critically ill types that accounted for most complications and mortalities associated with COVID-19. Although pathogenesis of COVID-19 has not been fully elucidated, there is consensus that immune-mediated inflammation plays an important role in the progression of this disease, just as it did in prior coronavirus infections (2). While an adequate immune response is essential for viral elimination, the over-activated host immune system may lead to immunopathology and clinical deterioration in many viral infections including COVID-19 (3). Intense systemic inflammatory response is one of the key features of severe COVID-19 patients, which is characterized by declined yet markedly activated lymphocytes, elevated inflammatory markers, and progressing coagulopathy (4–8). Many of these factors have been reported in various studies to be associated with increased mortality of COVID-19, included C-reactive protein, serum ferritin, IL-6, IP-10, MCP1, TNFα, d-dimer et al. (5, 6, 9, 10).

During the past year, various therapeutic approaches have been raised and administered. It is currently consensus that antivirals, if there is any, should be optimally administered at the initial phase of virus acquisition. However, the timing is difficult in practice, and candidates of antivirals are very limited. Therefore, control of the overactivated immune response at an earlier stage may provide a second chance. Immunomodulation therapy includes glucocorticoids, inflammation blockers, intravenous immunoglobulin, and convalescent plasma were used in various settings (8). The preliminary results from RECOVERY study established that low-dose dexamethasone administered in COVID-19 patients could lead to reduced 28-day mortality especially in those requiring oxygen therapy (11), indicating the benefit of appropriate inflammation control in the prognosis of COVID-19.

Intravenous immunoglobulin (IVIg) contains polyclonal immunoglobulin G isolated and pooled from healthy donors. The application of high-dose IVIg for its immunomodulatory functions could be traced back to 1981, when it was first used in refractory idiopathic thrombocytopenia (12). IVIg exhibits immunomodulatory capacities as demonstrated in many autoimmune or inflammatory diseases (13–16), as well as clinical benefits in prior coronavirus infections at a higher dose (17–19). Among studies of immunomodulators, we were the first to report use of high-dose IVIg as an immune modulation in deteriorating COVID-19 patients, and found that such therapy at an appropriate timing could prevent disease progression and improve the prognosis of severe COVID-19 patients (20). Following our report, several observational and interventional studies have been conducted to evaluate the efficacy of IVIg, including three published randomized trials (21–27). However, their results were controversial, probably due to variations in IVIg timing and dosing in different settings.

Given its efficacy in modulating immune inflammation and the overall safety profile, we consider high-dose IVIg an option at the early stage of deterioration in patents with COVID-19, and the timing of administration is critical for the prognosis. Early this year, we were the first to initiate a randomized controlled trials in China, to evaluate the benefit of high-dose IVIg in severe COVID-19 patients comparing with standard care (NCT04261426) (28). Unfortunately, the randomization was not carried out due to force majeure at that time in China. However, high-dose IVIg was used as planned in severe COVID-19 patients in participating centers. Here we retrospectively studied the efficacy of high-dose IVIg combined with standard care versus standard care only in patients with severe COVID-19.

## Methods

### Study Design and Participants

We conducted a multi-center retrospective cohort study in Jin-Yintan Hospital, Zhongnan Hospital of Wuhan University, and Sino-French campus of Tongji Hospital from February 7 to March 30, 2020 in Wuhan, China. The inclusion criteria were 1) age ≥ 18 years old, 2) confirmed diagnosis of SARS-CoV-2 infection and met any of the following criteria: respiratory distress (≥30 breaths/min); or oxygen saturation≤ 93% at rest; or arterial partial pressure of oxygen (PaO2)/fraction of inspired oxygen (FiO2) ≤ 300 mmHg (l mmHg=0.133 kPa), which graded as severe type according to the Chinese Recommendations for Diagnosis and Treatment of Novel Coronavirus (SARS-CoV-2) Infection (4^th^ version) (29), and 3) the interval between the onset of symptoms and hospitalization was within 2 weeks. The clinical symptoms mainly included fever, cough, dyspnea, diarrhea, and other related symptoms. Patients participating in other interventional clinical trials, or showing evidence of pneumonia caused by pathogens other than SARS-CoV-2 (including but not limited to influenza A virus, influenza B virus, bacterial pneumonia, fungal pneumonia, non-infectious causes, etc.), were excluded from the study.

The selected patients were enrolled and divided into two groups according to their treatment history: the IVIg group (high-dose IVIg therapy coupled with standard care following admission) and the control group (standard care only). Specifically, high-dose IVIg therapy (Shandong Taibang Biological Products Co., Ltd., Hualan Biological Engineering Inc., Chengdu Rongsheng Pharmaceuticals Co., Ltd; **Supplementary Table S1**) was defined as the total dose of 2 g per kilogram body weight, divided over 2–5 days. Standard care includes oxygen therapy, empirical antivirals (including one of the following, abidol, IFN-α, lopinavir/ritonavir, or ribavirin, as suggested by national and local recommendations), short course of glucocorticoids when considered necessary, and other supportive measures when needed. The clinical and laboratory variables, including the demographic information, clinical features, course of treatment, and laboratory results, were collected from electronic medical records of all patients.

The study protocol was approved by the institutional ethics board of Peking Union Medical College Hospital (PUMCH, No. ZS-2299, Feb 6, 2020), and all participants provided written consent for participating this study.

### Outcome Measures

The primary endpoint was the 28-day mortality in the study population. Secondary clinical outcomes included time to clinical improvement after admission, defined as a reduction of two points on the seven-category ordinal scale or live discharge from the hospital, clinical status as assessed with the seven-category ordinal scale on days 7, 14, and 28, the duration of mechanical ventilation, the duration of hospitalization in survivors, the duration of positive RT-PCR results, and the time to normalizations of inflammatory factors including interleukin (IL)-6, IL-8, IL-10, tumor necrosis factor (TNF)-α, hypersensitive C-reactive protein (hsCRP), ferritin, and erythrocyte sedimentation rate (ESR).

The seven-category ordinal scale consisted of the following categories: 1, not hospitalized with resumption of normal activities; 2, not hospitalized, but unable to resume normal activities; 3, hospitalized, not requiring supplemental oxygen; 4, hospitalized, requiring supplemental oxygen; 5, hospitalized, requiring nasal high-flow oxygen therapy and/or non-invasive mechanical ventilation; 6, hospitalized, requiring extracorporeal membrane oxygenation (ECMO) and/or invasive mechanical ventilation; and 7, death.

For the testing of inflammatory markers, the following instruments and reagents were used: IL-6 (up-converting phosphor assay, Beijing Rejing Biotechnology), IL-8/IL-10/TNF-α (bead-based immunoassay, MPXHCYTO-60K), hsCRP (turbidimetric inhibition immunoassay, MULTIGENT Vario), ferritin (chemiluminescence immunoassay, TaiGen Biotechnology), ESR (SD-100 Automated ESR Analyze, VES-TECH 20). Normal ranges of measured inflammatory markers in this study are as follows: IL-6 < 7.0 pg/ml, IL-8 < 62 pg/ml, IL-10 < 9.1 pg/ml, TNF-α < 8.1 pg/ml, hsCRP <10 mg/ml, serum ferritin < 400 ug/L for male and < 150 ug/L for female, ESR<15mm/h for male and <20 mm/h for female.

### Statistical Analysis

To address non-randomized treatment allocation and to correct for the difference in demographic and other clinical factors between the two groups, inverse probability of treatment weighting (IPTW) was performed. The propensity score is a conditional probability of having the particular exposure (high-dose IVIg therapy with standard care versus standard care only) given a set of baseline measured covariates which have been reported or might influence the prognosis. These variables included sex, age, comorbidities (hypertension, diabetes, chronic respiratory, and cardiac disease), disease onset days, disease grading based on the seven-category scale, use of other therapeutics including antivirals, glucocorticoids, and traditional medicine during disease course, baseline lymphocyte counts and platelet counts. After propensity scores were calculated, distributions of propensity scores in two groups before and after IPTW analysis were analyzed by Kernel density estimation. The propensity scores can be used as a covariate in adjusting the effect for baseline difference and can also be used in calculating inverse weights to estimates of the treatment effects in IPTW analysis. In the IPTW adjustment, weighting was performed as (1/propensity score) for IVIg group and [1/(1−propensity score)] for control group. Weights were used to estimate average treatment effects in treated patients and generate the IPTW group. For covariates with missing value which may cause bias, the multiple imputation (MI) was used to impute the missing laboratory results. MI was conducted using Bayesian methods in SPSS to generate five data sets and the synthesized complete case was applied for sensitive analysis (IPTW-MI model). Standardized differences were estimated for all the baseline covariates before and after IPTW to assess the balance. Although there is no universally agreed criterion as to what threshold of the standardized difference can be used to indicate important imbalance, it is commonly regarded that standardized differences of less than 10.0% for a given covariate indicate a relatively small imbalance (30, 31).

For variables following normal distribution, data were presented as mean and standard deviations and were analyzed by Student’s t-test. For variables following non-normal distribution, data were expressed as median and range and were compared by the Mann-Whitney U test. Differences in qualitative results were analyzed by the chi-square test or Fisher’s exact test where appropriate. Survival rates were analyzed using the Kaplan-Meier method and differences between the two groups were analyzed with the log-rank test. Univariate analysis was performed to identify prognostic variables related to overall survival and disease-free survival. Univariate variables with p values <0.05 were selected for inclusion in the multivariate Cox proportional hazard regression model. Adjusted hazard ratios (HR) along with the corresponding 95% confidence intervals (CI) were calculated. P<0.05 was considered statistically significant. All statistical analyses were performed using SPSS (version 25.0, SPSS Inc., Chicago, IL, USA) and R (version 3.2.4, R Foundation, Vienna, Austria).

## Results

### Patient Characteristics

**Figure 1:**
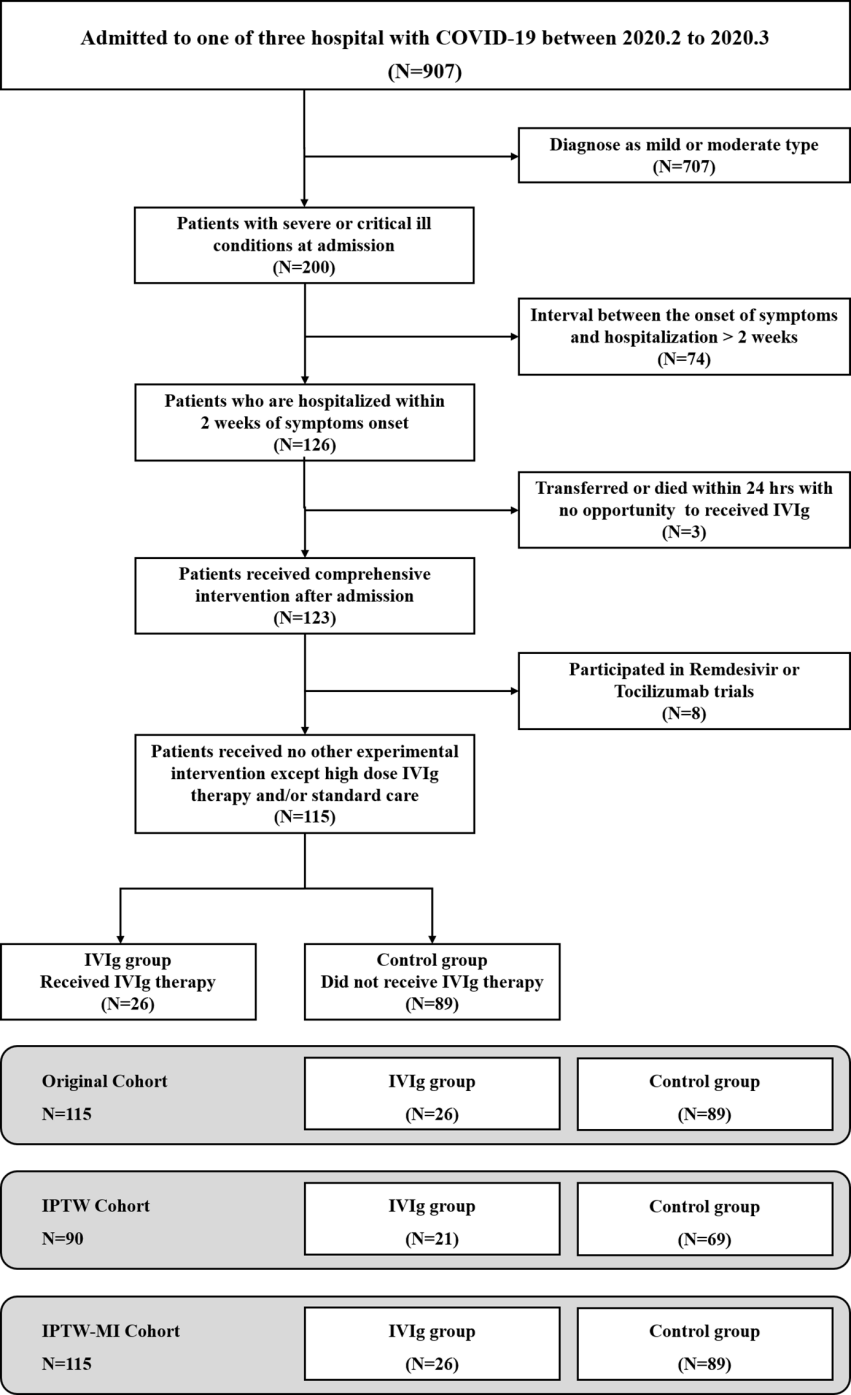
Selection and analysis process of the high-dose intravenous immunoglobulin (IVIg) and control groups.

**Figure 2:**
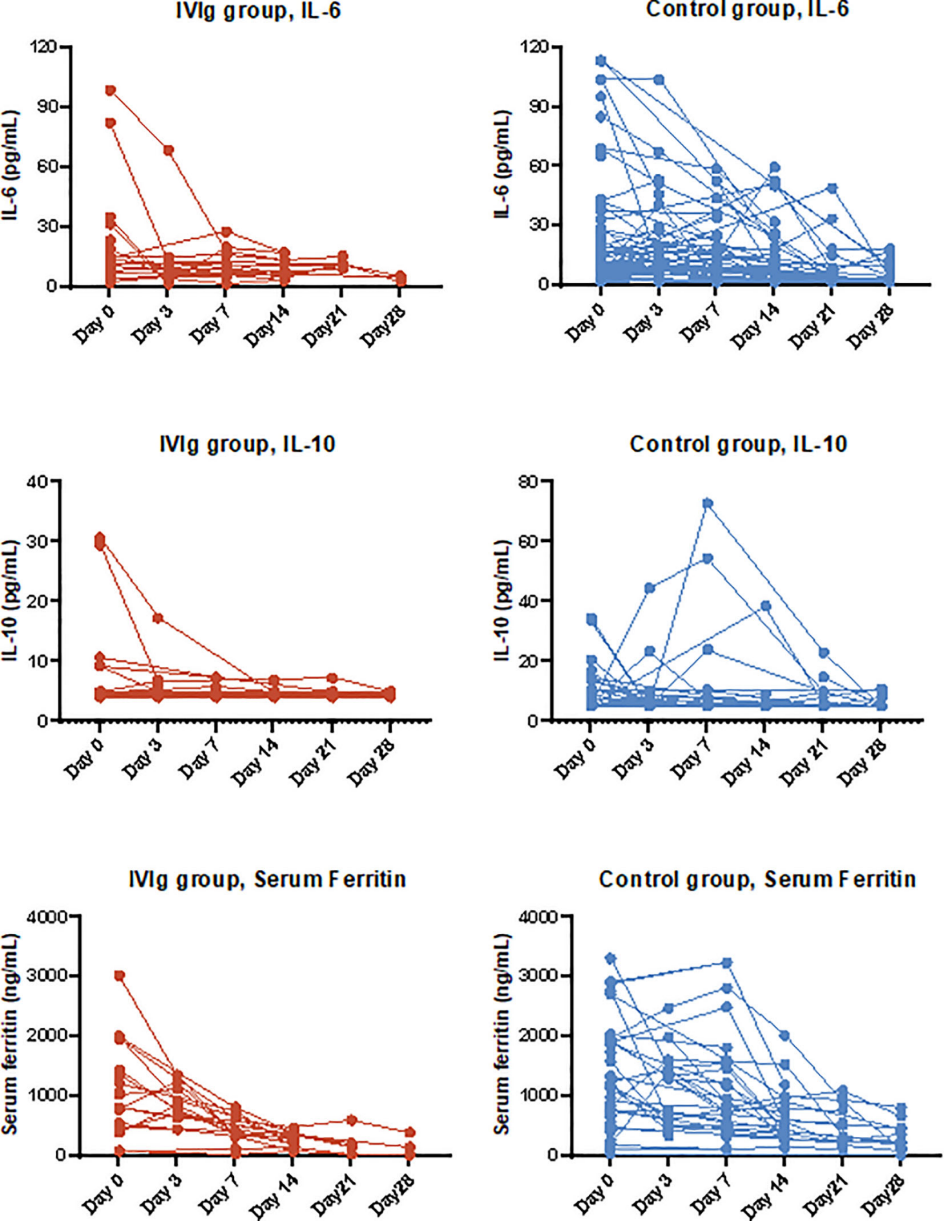
Dynamic of cytokines and inflammatory markers in severe hospitalized coronavirus disease 2019 (COVID-19) patients with high-dose intravenous immunoglobulin (IVIg) therapy and/or standard care.

A total of 907 patients with confirmed COVID-19 were available for screening from the three institutes. Of these patients, 200 were classified as severe or critically ill patients, and 115 were eligible to be included in the unadjusted analysis (**Figure 1**). Unadjusted patient characteristics were listed in **Table 1**. The IVIg group consisted of 26 patients. Their median age was 58.0 years old (IQR 42.2, 65.8), and 19 (73%) were men. Ten (38%) of them had hypertension, 2 (8%) had diabetes mellitus, and 5 (19%) had cardiac disorders. The most frequent symptoms included fever, cough, and dyspnea; the average level of pulse O_2_ saturation was 90% at ambient air on admission. Patients were admitted at 10 days (IQR 7, 12) of disease onset. Nineteen (73%) of them required supplemental oxygen therapy on admission, including one requiring non-invasive mechanical ventilation and one requiring invasive mechanical ventilation. The Murray lung injury score was 3.93 (SD 0.22) on admission. Patients also presented with slightly reduced lymphocyte count at 0.9×10^9^/L (IQR 0.62,1.50), and moderately elevated levels of inflammatory markers including IL-6, hsCRP, and ferritin as shown in **Table 1**. All patients in IVIg group received high-dose IVIg at an average of 13.2 (SD 6.6) days of disease onset. The course of high-dose IVIg was 5 (IQR 5, 9) days, with the total dose of 122.5 (IQR 95.0, 213.8) grams. After IVIg treatment, the lymphocytes increased and inflammatory cytokine declined in recovery patients (**Supplementary Figures S2**, **S3**, and **Figure 2**). In addition to IVIg, 22 (85%) patients received various regimens of empirical antiviral treatment, and 18 (69%) received glucocorticoids during hospitalization. The average course of glucocorticoid in the IVIg group was 7.0 (SD 3.5) days, with a total dose equivalent to methylprednisolone 296.3 (SD 155.8) mg, comparable with those of the control group.

**Table 1:** Clinical and relevant baseline characteristics of patients.

Characteristic	Total (n = 115)	IVIg group (n = 26)	Control group (n = 89)	SD (%)
**Demographics**				
Age, median (IQR)	59 (47–69)	58 (42–65)	59 (48–70)	16
Male sex, No. (%)	76 (66)	19 (73)	57 (64)	18
**Any comorbidity, No. (%)**				
Hypertension	52 (45)	10 (38)	42 (47)	9
Diabetes	24 (21)	2 (8)	22 (25)	39
Chronic cardiac disease	18 (16)	5 (19)	13 (15)	11
Chronic respiratory disease	11 (10)	2 (8)	9 (10)	4
**Any symptoms, No. (%)**				
Fever	111 (97)	22 (85)	89 (100)	31
Cough	97 (84)	22 (85)	75 (84)	8
Dyspnea	96 (83)	22 (85)	74 (83)	10
Fatigue or myalgia	52 (45)	9 (35)	43 (48)	19
Diarrhea	30 (26)	4 (15)	26 (29)	24
**Vital signs**				
Systolic BP, mm Hg	132 ± 19	131 ± 14	133 ± 20	5
HR,/min	88 (80–102)	85 (81–100)	89 (80–105)	9
RR, breaths/min	22 (20–25)	22 (20–23)	22 (20–25)	2
SPO_2_, %	90 (87–94)	91 (89–94)	89 (86–92)	6
**Complete blood count**				
WBCs, × 10^9^/L	6.59 (4.38–9.64)	6.25 (4.15–10.28)	6.60 (4.42–9.56)	4
LYM, × 10^9^/L	0.76 (0.57–1.02)	0.90 (0.62–1.05)	0.73 (0.56–0.91)	19
PLT, × 10^9^/L	191 (148–269)	172 (144–279)	199 (150–270)	18
**Inflammatory biomarkers**				
IL–6, pg/mL	13.0 (8.0–31.5)	15.5 (10.5–34.0)	11.0 (7.2–32.2)	15
IL–8, pg/mL	15.2 (6.0–27.3)	16.2 (5.8–23.8)	15.2 (5.9–28.4)	3
IL–10, pg/mL	5.2 (5.0–10.3)	5.0 (5.0–8.5)	5.5 (5.0–11.2)	2
hsCRP, mg/mL	48 (17–94)	28 (7–91)	45 (17–101)	14
Ferritin, ng/mL	807.0 (473.4–1383.3)	774.1 (444.2–1525.4)	838.8 (501.5–1351.1)	18
ESR, mm/h	50 (25–65)	35 (19–67)	50 (27–65)	14
**Immunoglobulins**				
IgA, g/L	2.2 (1.8–3.0)	2.7 (1.9–4.6)	2.2 (1.6–3.0)	2
IgG, g/L	11.1 (9.0–13.1)	11.8 (8.5–15.7)	11.1 (9.3–13.1)	2
IgM g/L	1.0 (0.7–1.2)	1.0 (0.9–1.1)	0.9 (0.7–1.3)	3
**Seven–category scales at admission**	4.0 ± 0.67	3.85 ± 0.67	4.06 ± 0.66	26
3, No. (%)	20 (17)	7 (27)	13 (15)	24
4, No. (%)	79 (69)	17(65)	62 (70)	7
5, No. (%)	11 (10)	1 (4)	10 (11)	25
6, No. (%)	5 (4)	1 (4)	4 (4)	3
**Murray lung injury scores at admission**	3.93 ± 0.22	3.90 ± 0.28	3.94 ± 0.20	11
**Time to admission after onset, median (IQR)**	10 (7–12)	10 (7–12)	10 (8–12)	5
Earlier (< 7 days), No. (%)	29 (25)	7 (27)	22 (25)	12
Later (between 7–14 days), No. (%)	86 (75)	19 (73)	67 (75)	12
**Treatment during hospitalization, No. (%)**				
Antiviral treatment	100 (87)	22 (85)	78 (88)	7
Arbidol	75 (65)	15 (58)	60 (67)	16
IFN–α	34 (30)	14 (54)	20 (22)	53
LPV/r	31 (27)	9 (35)	22 (25)	17
RBV	12 (10)	3 (12)	9 (10)	4
OSV	33 (29)	8 (31)	25 (28)	5
Antibiotic treatment	100 (87)	25 (96)	75 (84)	37
Moxifloxacin	73 (63)	15 (58)	58 (65)	12
Cefoperazone and tazobactam	47 (41)	13 (50)	34 (38)	19
Antifungal treatment	11 (10)	5 (19)	6 (7)	29
TCM	58 (50)	15 (58)	43 (48)	15
LMWH	19 (16)	4 (15)	15 (17)	3
Glucocorticoids	71 (62)	18 (69)	53 (60)	17
**IVIg therapy**	26 (23)	26 (100)	0 (0)	—
Initiation time, days, median (IQR)	—	2 (1–4)	—	—
Course, days, median (IQR)	—	5 (5–9)	—	—
Amount, g, median (IQR)	—	122.5 (95.0–213.8)	—	

SD, standard deviation; Tmax, maximal temperature; BP, blood pressure; HR, heart rate; RR, respiratory rate; SPO_2_, pulse oximeter O2 saturation; WBC, white cell counts; LYM, lymphocyte counts; PLT, platelet counts; IL, interleukin; CRP, C-reactive protein; ESR, erythrocyte sedimentation rate; IFN-α, Interferon-alfa; LPV/r, lopinavir/ritonavir; RBV, ribavirin; OSV, oseltamivir; TCM, traditional Chinese medicine; LMWH, low molecular weight heparin.

The control group included 89 patients with severe COVID-19, who did not receive treatment with IVIg. Differences of baseline and clinical characteristics were listed in **Table 1**. To correct for the potential imbalances, we performed IPTW and IPTW-MI respectively as described, and the results were shown in **Supplementary Table S3** and **Supplementary Figure S1**. Most of the baseline characteristics were balanced between the IVIg and control groups after adjustment.

**Table 2** shows the clinical outcomes of all included COVID-19 patients before adjustment. At 28 days of disease onset, one patient (4%) in IVIg group and 25 patients (28%) in the control group died as a result of disease progression. With univariant analysis, the 28-day mortality rate of IVIg group was lower than that of the control group (HR 0.12, 95% CI 0.02–0.88, p=0.037). At 7, 14, and 21 days of follow-up, patients with clinical improvement, as measured by the proportions of patients with improved seven-category ordinal scale (decline larger than 2 scores) was 8, 35, and 62% in the IVIg group, and 0, 31, and 46% in the control group, respectively. The statistical difference was significant between the two groups at day 7 after admission, suggesting the obvious clinical benefit of IVIg (**Table 3**). The length of hospitalization in the IVIg group was 18 days *vs*. 24 days in the control group (HR 1.23, 95% CI 0.75–2.00), and no difference was observed in time to RT-PCR conversion. There was a tendency of shortened invasive ventilation time in the IVIg group, but the difference was not significant. In addition, patients in the IVIg group generally required less time to achieve normalization of inflammatory markers such as IL-6, IL-10, and ferritin. Specifically, time to IL-6 normalization in the IVIg group was much shorter than that of the control (6 *vs*. 11 days, HR 3.11, 95% CI 1.33–7.26).

**Table 2:** Clinical outcomes of patients before the propensity score adjustment.

Outcomes	IVIg group (n=26)	Control group (n=89)	Absolute difference (95% CI)	*P* value
**Primary outcome**				
28 days mortality, no. (%)	1 (4%)	25 (28%)	−24% (−36 to −12%)	<0.001
**Secondary outcomes**				
Time to clinical improvement, days (IQR)	15 (12–23)	18 (12–22)	0 (−4 to 4)	0.853
Clinical improvement, no. (%)				
Day 7	2 (8%)	0 (0%)	8% (2 to 13%)	0.001
Day 14	9 (35%)	28 (31%)	3% (−18 to 24%)	0.764
Day 21	16 (62%)	41 (46%)	15% (−7 to 38%)	0.168
Day 28	22 (85%)	51 (57%)	27% (6 to 48%)	0.003
Duration of mechanical ventilation, days (IQR)	10 (3–13)	9 (3–18)	1 (−11 to 13)	0.853
Duration of hospitalization in survivors, days (IQR)	18 (15–26)	24 (15–29)	−2 (−4 to 1)	0.597
Duration of positive RT-PCR results, days (IQR)	10 (7–18)	10 (5–6)	2 (−3 to 6)	0.099
Time to inflammatory markers normalization, days (IQR)				
IL6	6 (4–11)	11 (7–18)	−4 (−10 to −2)	0.051
IL8	4 (2–6)	5 (2–12)	−4 (−8 to 1)	0.113
IL-10	5 (2–7)	7 (4–12)	−4 (−8 to 1)	0.020
TNFα	5 (4–24)	9 (5–18)	−3 (−17 to 7)	0.599
hsCRP	11 (7–21)	14 (9–18)	−3 (−6 to 4)	0.684
Serum ferritin	6 (5–7)	12 (7–21)	−7 (−12 to −3)	0.002
ESR	11 (8–12)	13 (6–24)	−4 (−12 to 3)	0.197

**Table 3:** Multivariable cox regression analysis of 28 days mortality of patients receiving high-dose intravenous immunoglobulin (IVIg).

Outcomes	IVIg	Yes					
	No	Unadjusted model		Demographic-adjusted model*		Fully adjusted model*	
			
		HR (95.0% CI)	P value	HR (95.0% CI)	P value	HR (95.0% CI)	P value
Original cohort	Reference	0.12 (0.02–0.88)	0.037	0.14 (0.02–1.06)	NS	0.26 (0.03–1.24)	NS
IPTW model	Reference	0.08 (0.02–0.26)	<0.001	0.08 (0.02–0.26)	<0.001	0.24 (0.06–0.99)	0.048
IPTW-MI model	Reference	0.11 (0.05–0.27)	<0.001	0.11 (0.06–0.32)	<0.001	0.27 (0.10–0.57)	0.031

*Demographic-adjusted: controlled for age and sex as covariates; fully adjusted: controlled for age, sex, comorbidity, disease onset days, baseline seven scale category, the use of arbidol, LPV/r, IFN, RBV, OSV, antibiotics, antifungals, TCM, glucocorticoids, LMWH as covariates.

The IPTW adjustment resulted a balanced population of patients between the IVIg group and the control group (**Supplementary Table S2** and **Supplementary Figure S1**) and the efficacy evaluation following IPTW adjustment was also significant (**Supplementary Table S4**). A similar difference between the two groups was observed in 28-day mortality, with 3% in the IVIg group and 27% in the control group (OR 0.08, 95% CI 0.02–0.26), and the time to clinical improvement was also numerically shorter in the IVIg group. The estimated duration of hospitalization was 18 and 25 days in the IVIg and control group, respectively. Similarly, the time to inflammatory maker normalization was much shorter in the IVIg group compared with that of the control group in IPTW and IPTW-MI model, most prominent in IL-6 (4 *vs*. 11 days) and ferritin (6 *vs*. 12 days) (**Supplementary Tables S4** and **S5**, **Figure 2**).

### Overall Patient Survival With Severe COVID-19

**Figure 3:**
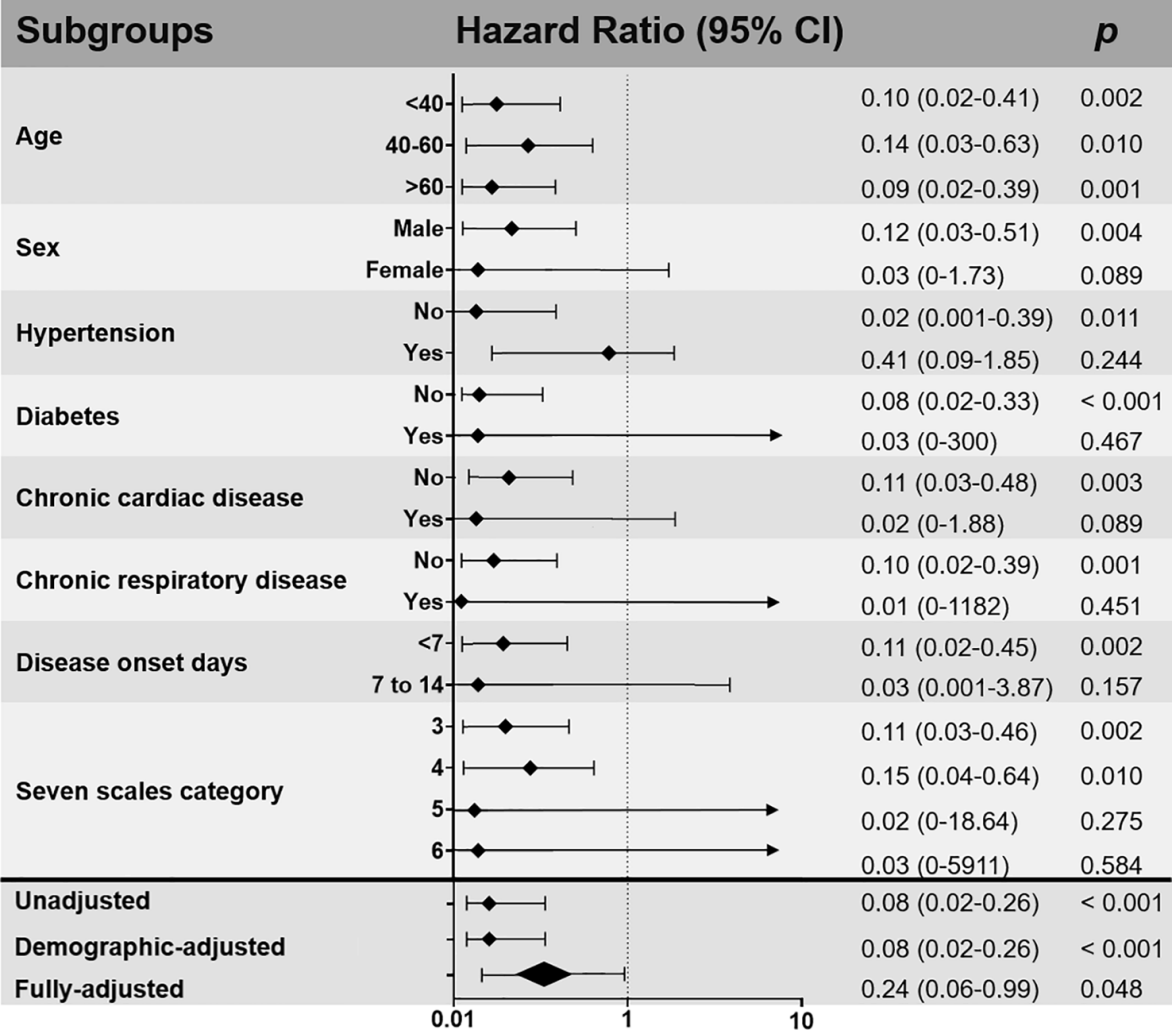
Subgroup and multivariable analyses of the impact of intravenous immunoglobulin (IVIg) therapy on 28-day mortality after inverse probability of treatment weighting (IPTW) adjusted. Demographic-adjusted: controlled for age and sex as covariates; fully adjusted: controlled for age, sex, comorbidity, disease onset days, baseline seven scale category, the use of arbidol, LPV/r, IFN, RBV, OSV, antibiotics, antifungals, TCM, glucocorticoids, LMWH as covariates.

**Figure 4:**
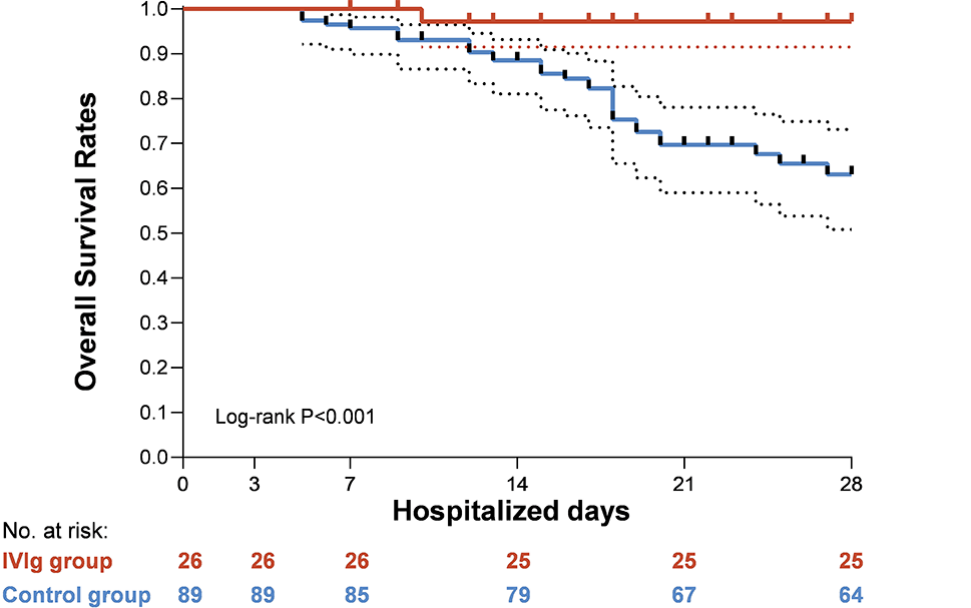
Kaplan-Meier curves for overall survival in severe hospitalized coronavirus disease 2019 (COVID-19) patients with high-dose intravenous immunoglobulin (IVIg) therapy and/or standard care.

Multivariable cox regression was done and the results was shown in **Table 3** and **Figure 3**. Use of high-dose IVIg was strongly associated with reduced 28-day mortality in both IPTW and IPTW-MI analysis. With the fully adjusted model that has been controlled for demographic factors and use of different treatments, the HR of 28-day mortality in the high-dose IVIg group was 0.24 (95% CI 0.06–0.99, p<0.001) in the IPTW adjustment, and 0.27 (95% CI 0.10–0.57, p=0.031) in IPTW-MI model. The Kaplan–Meier estimate of 28-day survival before and after adjustments was shown in **Figure 4**. In these three models, timely use of high-dose IVIg was associated significantly reduced mortality.

In subgroup analysis stratified by various factors (**Figure 3**), patients receiving high-dose IVIg within 1 week of disease onset were associated with reduced 28-day mortality rate (HR 0.11, 95% CI 0.02–0.45, p=0.002) compared to those started in the second week of infection (HR 0.03, 95% CI 0.001–3.87, P=0.157). In addition, it seems that patients with absence of co-morbidities tend to benefit more from the use of high-dose IVIg (**Figure 3**).

### Safety

No adverse event (AE) was reported among the 26 patients with severe COVID-19 who were treated by high-dose IVIg. Across the excluded 51 patients with common type COVID-19 who were treated with at least once IVIg therapy, only three patients (5.9%) experienced AE that included palpitation (n=1), dizziness (n=1), and rash (n=1) at the end of infusion. Those AEs were transient and did not require the cessation of IVIg therapy.

## Discussion

Since the outbreak of COVID-19, tremendous global efforts have been devoted to developing effective treatment against this novel virus and its related disease. Unfortunately, limited therapeutic approach has proved efficacious up to date. Systemic corticosteroids proved efficacious in certain studies, but its impact on host immunity, the risk of subsequent infections and long-term effect remain unclear (11, 32). Remdesivir was also suggested in treating patients who require supplemental oxygen but dispense with high-flow device, non-invasive and invasive mechanical ventilation, or ECMO (33). However, remdesivir provided little benefit in populations other than hypoxic patients. In addition, its availability and price remain thorny issues for most countries. Based on the clinical understanding of this novel disease, we have raised high-dose IVIg as a possible solution to deteriorating patients with COVID-19, and reported three cases successfully treated with high-dose IVIg (20). Here, we reported 26 patients with severe COVID-19 who were treated by high-dose IVIg in addition to standard care, in comparison with those using standard care only. Overall, we found that administration of high-dose IVIg at an early phase of disease deterioration was associated with markedly reduced mortality, quicker normalization of inflammatory status, and improved clinical outcomes in COVID-19.

Abundant real-world experience has demonstrated that infection of SARS-CoV-2 could be quite heterogenous. While the majority of infected individuals present as mild or moderate types with relatively benign recovery, around 20% infected patients may progress to more severe and critical types with higher risks of mortality. Moreover, the clinical events leading to unfavorable outcomes include not only the collapse of lung tissues, but also the associated fulminant systemic inflammation and coagulation disorders. The deterioration of COVID-19 usually takes place after one to two weeks of disease onset, associated with a continuous decreasing lymphocyte count and significant elevation of neutrophils, as well as markedly elevated inflammatory markers included C-reactive protein, serum ferritin, IL-6, IP-10, MCP1, TNFα, d-dimer et al. (9, 10). With limited choice of antivirals, control of the overactivated immune response at an earlier stage may provide a second chance.

IVIg is a blood preparation isolated and concentrated from healthy donors mainly consisting of IgG, and has been used in clinical practice for many years. While regular small dose of IVIg mainly serves as substitutive therapy for primary or acquired immunodeficiencies, high-dose IVIg has been used for immune modulation and anti-inflammation under many clinical settings (35). This effect was partially proved in prior studies with other severe acute viral pneumonia (36). As regarding COVID-19, more results have been reported following our first cases series (20). Another case series in Iran reported similar effect of high-dose IVIg from five patients with severe COVID-19 who failed standard treatment at that time (37). A retrospective study in China compared outcomes of severe and critical COVID-19 patients with different timing of high-dose IVIg treatment, and found that IVIg initiation within 48h of ICU stay was associated with decreased use of mechanical ventilation, shortened ICU and hospital stay, and reduced 28-day mortality (23.3% within 48h and 57.1% after 48h respectively) (22). Recently, three randomized trials of COVID-19 have come out using high-dose IVIg. Sakoulas et al. reported that IVIg 0.5g/kg/d combined with methylprednisolone 40 mg for 3 days, compared with standard therapy, could reduce hospital stay and progression to mechanical ventilation in hypoxic COVID-19 patients (25). Gharebaghi et al. showed that patients who failed initial treatment could receive additional clinical benefit from IVIg with significantly reduced mortality rate (26). However, another Iran study only observed a potential reduced ICU stay among the survivors receiving 0.4g/kg/d IVIg for 3 days, with no significant impact on mortality (27). Such controversial results were largely due to varying timing and dosage of IVIg therapy. Few studies provided definite information of patients’ disease course. In some cases, regular supplemental doses of IVIg were used to enhance passive immunity, rather than high-doses to regulate the immune inflammation. These factors may all interfere with the explanation of study results.

In our study, most patients were hospitalized and started on high-dose IVIg within 2 weeks of disease onset. Subgroup analysis showed an additional benefit when IVIg was administered within the first week of infection, in accordance with our deduction that the timing of treatment is most critical for COVID-19 patients’ prognosis and management (8). IVIg at a total dose of 2 g per kg weight was selected in our study for anti-inflammation, based on the well-established practice in immune modulation therapy using IVIg for other diseases (38, 39). Considering the potential cardiac or renal impairment in severe COVID-19 patients, we actually modified the dose into 0.3–0.5g/kg/day for 5 days as a safe and potent regimen. It would also be worthwhile to try to dispense the total dose to even a shorter course of treatment in the future, since the daily dose may have an impact on the efficacy. There were several patients who received prolonged course of IVIg after completing the required regimen for other purposes such as antibacterial et al. Our results showed that high-dose IVIg administered at appropriate time point and proper dosage was associated with reduced 28-day mortality and improved clinical outcome, with patients treated earlier even more survival benefit compared with those treated later.

The safety data from our patients indicated that the application of high-dose IVIg was well-tolerable. The frequency and type of mild AEs were also in conformity with previous reports. The majority of side effects during IVIg infusion were mild and transient, which usually alleviated after infusion withdrawal. Although IVIg-related thrombotic complications were not observed in our patients, clinicians need to be vigilant about the risk of thromboembolic events as both COVID-19 and IVIg therapy might predispose to these events.

We have recently reviewed the potential mechanisms contributing to the immunomodulatory effects of IVIG in viral pneumonia including COVID-19 (36). Unlike low-dose of IVIg that exhibits proinflammatory activity through complement activation or Fc fragment binding, high concentration of IVIg has anti-inflammatory properties. As current batches of IVIg lack cross-neutralizing antibodies against SARS-CoV-2 (40), several non-specific mechanisms may have contributed to the control of COVID-19 by exerting anti-inflammatory effect on cytokine network and on innate as well as adaptive immune system (24, 36). High-dose IVIg could modulate the activation of cytokine network, neutralize autoantibodies, and regulate proliferation of immune cells (41).

It was a pity that we were not able to conduct the randomized trial in the real practice as arranged, which is the biggest limitation of the present study. We are fully aware that randomized clinical trials would be much more powerful in evaluating the efficacy of high-dose IVIg in treating severe and critical COVID-19 patients. Therefore, we used IPTW and IPTW-MI adjustment to maximally balance the confounding factors that may exert an effect on the outcomes of COVID-19 patients. A second limitation is that our analysis excluded patients who were treated with remdesivir, which has been considered as part of the “standard of care” in some countries. As remdesivir has not accessed approved or emergency use authorization by China National Medical Products Administration, enrollment in clinical trials is the primary way to access remdesivir. At the time of our study (Feb to March 2020), we have excluded three patients using remdesivir/placebo (two in IVIg group and one in control group) and five patients using tocilizumab (two in IVIg group and three in control group). These patients did survive after 30 days of admission, however, due to the small number and masking design of patients, it was not feasible to do stratified analysis. In order to avoid the trial bias caused by the excessive subjectiveness of the patients and the clinicians, we excluded these patients though it might affect the external validity. Another limitation is that we were not able to carry out the T cells subgroup analysis in these designated centers during the study. It is noted that an increased release of pro-inflammatory cytokines by Th1 and Th17 cells (e.g., IL-6 and IL-17) was observed in COVID-19 patients, associated with the hyperinflammatory conditions (42, 43), high-dose IVIg has been shown to inhibit the activation, and subsequent production of cytokines by Th1 and Th17 cells in several clinical studies and *in vitro* experiments, and further reconstruct the balance between Th1, Th2, Th17, and Treg cells (44–47). It would be interesting to follow the dynamics and functions of CD4^+^ T cell subsets along with application of high-dose IVIg to better understanding the mechanism underlying COVID-19.

In conclusion, the present study reported results of high-dose IVIg use in patients with severe COVID-19. Of note, the most important points in our recommendation of using IVIg would be the right dose and the appropriate timing, which requires for continuous and close monitor of affected patients. Our results demonstrated that high-dose IVIg administered in severe COVID-19 patients within 14 days of onset was linked to improved survival rate in this population. This effect may be more prominent with people having few comorbidities or treated at a relatively early stage of disease progression. There have been several randomized clinical trials registered and initiated recently. Their results are probably anticipated later this year, which will further build on our knowledge of high-dose IVIg regimen in COVID-19.

## Data Availability Statement

The original contributions presented in the study are included in the article/**Supplementary Material**. Further inquiries can be directed to the corresponding author.

## Ethics Statement

The studies involving human participants were reviewed and approved by Peking Union Medical College Hospital (PUMCH, No. ZS-2299, Feb 6, 2020). The patients/participants provided their written informed consent to participate in this study.

## Author Contributions

TL and WC designed the study. WC and XL drafted the manuscript. XL and YZ carried out the data processing and statistical analysis. WC, KH, ZM, YX, ZL, LR, and TL cared for the enrolled patients and collected all the clinical data. LL and YH reviewed the literature and revised the manuscript. All authors contributed to the article and approved the submitted version.

## Conflict of Interest

The authors declare that the research was conducted in the absence of any commercial or financial relationships that could be construed as a potential conflict of interest.
