# Corrigendum: High-Dose Intravenous Immunoglobulin in Severe Coronavirus Disease 2019: A Multicenter Retrospective Study in China

**DOI:** 10.3389/fimmu.2021.671443

**Published:** 2021-03-22

**Authors:** Wei Cao, Xiaosheng Liu, Ke Hong, Zhiyong Ma, Yuelun Zhang, Ling Lin, Yang Han, Yong Xiong, Zhengyin Liu, Lianguo Ruan, Taisheng Li

**Affiliations:** ^1^ Department of Infectious Diseases, Peking Union Medical College Hospital, Peking Union Medical College and Chinese Academy of Medical Sciences, Beijing, China; ^2^ Department of Basic Medical Sciences, School of Medicine, Tsinghua University, Beijing, China; ^3^ Tsinghua-Peking Center for Life Sciences, Beijing, China; ^4^ Department of Infectious Diseases, JinYin-tan Hospital, Wuhan, China; ^5^ Department of Infectious Diseases, Zhongnan Hospital of Wuhan University, Wuhan, China; ^6^ Medial Research Center, Peking Union Medical College Hospital, Peking Union Medical College and Chinese Academy of Medical Sciences, Beijing, China

**Keywords:** COVID-19, high-dose intravenous immunoglobulin, immunomodulation, 28-day mortality, inflammatory markers

## Error in Table

In the original article, there was a mistake in **Table 1** as published. The author identified the calculating errors in the percentage of comorbidity, symptoms, and treatment. The corrected **Table 1** appears below.

**Table 1:** Clinical and relevant baseline characteristics of patients.

Characteristic	Total (n = 115)	IVIg group (n = 26)	Control group (n = 89)	SD (%)
**Demographics**				
Age, median (IQR)	59 (47–69)	58 (42–65)	59 (48–70)	16
Male sex, No. (%)	76 (66)	19 (73)	57 (64)	18
**Any comorbidity, No. (%)**				
Hypertension	52 (45)	10 (38)	42 (47)	9
Diabetes	24 (21)	2 (8)	22 (25)	39
Chronic cardiac disease	18 (16)	5 (19)	13 (15)	11
Chronic respiratory disease	11 (10)	2 (8)	9 (10)	4
**Any symptoms, No. (%)**				
Fever	111 (97)	22 (85)	89 (100)	31
Cough	97 (84)	22 (85)	75 (84)	8
Dyspnea	96 (83)	22 (85)	74 (83)	10
Fatigue or myalgia	52 (45)	9 (35)	43 (48)	19
Diarrhea	30 (26)	4 (15)	26 (29)	24
**Vital signs**				
Systolic BP, mm Hg	132 ± 19	131 ± 14	133 ± 20	5
HR,/min	88 (80–102)	85 (81–100)	89 (80–105)	9
RR, breaths/min	22 (20–25)	22 (20–23)	22 (20–25)	2
SPO_2_, %	90 (87–94)	91 (89–94)	89 (86–92)	6
**Complete blood count**				
WBCs, × 10^9^/L	6.59 (4.38–9.64)	6.25 (4.15–10.28)	6.60 (4.42–9.56)	4
LYM, × 10^9^/L	0.76 (0.57–1.02)	0.90 (0.62–1.05)	0.73 (0.56–0.91)	19
PLT, × 10^9^/L	191 (148–269)	172 (144–279)	199 (150–270)	18
**Inflammatory biomarkers**				
IL–6, pg/mL	13.0 (8.0–31.5)	15.5 (10.5–34.0)	11.0 (7.2–32.2)	15
IL–8, pg/mL	15.2 (6.0–27.3)	16.2 (5.8–23.8)	15.2 (5.9–28.4)	3
IL–10, pg/mL	5.2 (5.0–10.3)	5.0 (5.0–8.5)	5.5 (5.0–11.2)	2
hsCRP, mg/L	48 (17–94)	28 (7–91)	45 (17–101)	14
Ferritin, ng/mL	807.0 (473.4–1383.3)	774.1 (444.2–1525.4)	838.8 (501.5–1351.1)	18
ESR, mm/h	50 (25–65)	35 (19–67)	50 (27–65)	14
**Immunoglobulins**				
IgA, g/L	2.2 (1.8–3.0)	2.7 (1.9–4.6)	2.2 (1.6–3.0)	2
IgG, g/L	11.1 (9.0–13.1)	11.8 (8.5–15.7)	11.1 (9.3–13.1)	2
IgM g/L	1.0 (0.7–1.2)	1.0 (0.9–1.1)	0.9 (0.7–1.3)	3
**Seven–category scales at admission**	4.0 ± 0.67	3.85 ± 0.67	4.06 ± 0.66	26
3, No. (%)	20 (17)	7 (27)	13 (15)	24
4, No. (%)	79 (69)	17(65)	62 (70)	7
5, No. (%)	11 (10)	1 (4)	10 (11)	25
6, No. (%)	5 (4)	1 (4)	4 (4)	3
**Murray lung injury scores at admission**	3.93 ± 0.22	3.90 ± 0.28	3.94 ± 0.20	11
**Time to admission after onset, median (IQR)**	10 (7–12)	10 (7–12)	10 (8–12)	5
Earlier (< 7 days), No. (%)	29 (25)	7 (27)	22 (25)	12
Later (between 7–14 days), No. (%)	86 (75)	19 (73)	67 (75)	12
**Treatment during hospitalization, No. (%)**				
Antiviral treatment	100 (87)	22 (85)	78 (88)	7
Arbidol	75 (65)	15 (58)	60 (67)	16
IFN–α	34 (30)	14 (54)	20 (22)	53
LPV/r	31 (27)	9 (35)	22 (25)	17
RBV	12 (10)	3 (12)	9 (10)	4
OSV	33 (29)	8 (31)	25 (28)	5
Antibiotic treatment	100 (87)	25 (96)	75 (84)	37
Moxifloxacin	73 (63)	15 (58)	58 (65)	12
Cefoperazone and tazobactam	47 (41)	13 (50)	34 (38)	19
Antifungal treatment	11 (10)	5 (19)	6 (7)	29
TCM	58 (50)	15 (58)	43 (48)	15
LMWH	19 (16)	4 (15)	15 (17)	3
Glucocorticoids	71 (62)	18 (69)	53 (60)	17
**IVIg therapy**	26 (23)	26 (100)	0 (0)	—
Initiation time, days, median (IQR)	—	2 (1–4)	—	—
Course, days, median (IQR)	—	5 (5–9)	—	—
Amount, g, median (IQR)	—	122.5 (95.0–213.8)	—	

SD, standard deviation; Tmax, maximal temperature; BP, blood pressure; HR, heart rate; RR, respiratory rate; SPO_2_, pulse oximeter O2 saturation; WBC, white cell counts; LYM, lymphocyte counts; PLT, platelet counts; IL, interleukin; CRP, C-reactive protein; ESR, erythrocyte sedimentation rate; IFN-α, Interferon-alfa; LPV/r, lopinavir/ritonavir; RBV, ribavirin; OSV, oseltamivir; TCM, traditional Chinese medicine; LMWH, low molecular weight heparin.

The authors apologize for this error and state that this does not change the scientific conclusions of the article in any way. The original article has been updated.

