# Supplementary material for: High-Dose Intravenous Immunoglobulin in Severe Coronavirus Disease 2019: A Multicenter Retrospective Study in China
Source: Front Immunol. 2021 Feb 19;12:627844. doi: 10.3389/fimmu.2021.627844 (PMC7933558; doi:10.3389/fimmu.2021.627844)
Supplement: Supplementary file 1 [file DataSheet_1.docx]

**Additional Information**

**Table S1. The formula of IVIg product from public patent application and journal publication**

| **Manufacture** | **IgA**  **(μg/mL)** | **IgD**  **(ng/mL)** | **IgE**  **(ng/mL)** | **IgM**  **(μg/mL)** | **IgG**  **(g/mL)** | **IgG subgroups (%)** | | | |
| --- | --- | --- | --- | --- | --- | --- | --- | --- | --- |
|  |  |  |  |  |  | **IgG1** | **IgG2** | **IgG3** | **IgG4** |
| **Taibang^[1,2]^**  **IVIg (n=4)** | 4.8±6.3 | 11.9±9.2 | 0.4±0.6 | 0.41±0.55 | 0.047±0.003 | 58.5±2.2 | 32.6±3.3 | 6.2±0.9 | 2.7±1.0 |
| **Rongsheng^[3]^**  **IVIg (n=3)** | 19.6±2.1 | NA | NA | NA | 0.049±0.016 | 55.1±1.6 | 38.7±6.6 | 7.1±1.0 | 2.5±0.5 |
| **Hualan^[4]^**  **IVIg (n=3)** | 5.3±0.3 | NA | NA | NA | 0.049±0.002 | 68.2±4.5 | 21.9±4.5 | 8.9±0.5 | 1.0±0.2 |
| **OctapharmaIVIg (n=6)** | 120.3±52.5 | 207.8±23.9 | 93.7±17.8 | 4.7±5.0 | 0.048±0.003 | 60.4±2.1 | 31.5±1.8 | 6.4±0.5 | 1.7±0.5 |
| **P value^*^** | 0.001 | <0.001 | <0.001 | 0.109 | 0.684 | 0.345 | 0.623 | 0.655 | 0.167 |

NA: Not available.

*: P value was calculated to compare the IVIg product of Taibang and Octapharma from Reference [1,2].

Reference:

[1] Method for preparing human immunoglobulin for intravenous injection, by C.Y., L.B., Y.C., et al. (2015). Patent CN104402993A. Available at: <https://patents.google.com/patent/CN104402993A/zh>.

[2] Method for preparing human immunoglobulin for intravenous injection, by C.Y., L.B., Y.C., et al. (2017). Patent CN104402993B. Available at: <https://patents.google.com/patent/CN104402993B/zh>.

[3] Method for preparing human immunoglobulin, by Y.H., L.J., L.H., et al. (2013). Patent CN102532307B. Available at: <https://patents.google.com/patent/CN102532307B/zh>.

[4] L.Y., Z.J., L.G., et al. Comparative study on the quality of intravenous injection human immunoglobulin products prepared by different processes. ***International journal of biologicals*** 42 (3) 129-133. DOI: 10.3760/cma.j.issn.1673-4211.2019.03.007 (2019).

**Table S2. Variables and proportions of missing data**

| **Characteristics** | **IVIg group (n=26)** | | | **Control group (n=89)** | | |
| --- | --- | --- | --- | --- | --- | --- |
|  | **Available** | **Missing** | **Missing %** | **Available** | **Missing** | **Missing %** |
| **Age** | 26 | 0 | 0 | 89 | 0 | 0 |
| **Sex** | 26 | 0 | 0 | 89 | 0 | 0 |
| **Comorbidity** | 23 | 3 | 11.5% | 85 | 4 | 4.5% |
| **Seven-category scales** | 26 | 0 | 0 | 89 | 0 | 0 |
| **Disease onset days** | 26 | 0 | 0 | 89 | 0 | 0 |
| **Treatment** | 26 | 0 | 0 | 89 | 0 | 0 |
| **LYM count** | 22 | 4 | 15.4% | 79 | 10 | 11.2% |
| **PLT count** | 22 | 4 | 15.4% | 86 | 3 | 3.4% |

Variables for calculating Propensity Scores included sex, age, comorbidities (hypertension, diabetes, chronic respiratory and cardiac disease), disease onset days, disease grading based on the seven-category scale, use of other therapeutics including antivirals, glucocorticoids and traditional medicine during disease course, baseline lymphocyte counts and platelet counts.

**Table S3. Clinical characteristics and treatment of patients after IPTW adjustment**

| **Patient characteristics** | **IVIg group***  **(n=114)** | **Control group**  **(n=115)** | **SD**  **(%)** |
| --- | --- | --- | --- |
| **Demographics** |  |  |  |
| **Age** | 60 (46-66) | 59 (45-69) | 1 |
| < 40, No. (%) | 25 (22) | 19 (17) | 5 |
| 40-60, No. (%) | 44 (39) | 46 (40) | 1 |
| 61-80, No. (%) | 39 (34) | 42 (37) | 2 |
| >80, No. (%) | 6 (5) | 8 (7) | 2 |
| **Sex** |  |  |  |
| Male, No. (%) | 86 (75) | 77 (67) | 8 |
| **Comorbidity, No. (%)** |  |  |  |
| Hypertension | 50 (44) | 50 (43) | 0 |
| Diabetes | 20 (18) | 22 (19) | 2 |
| Chronic cardiac disease | 12 (11) | 16 (14) | 3 |
| Chronic respiratory disease | 4 (4) | 10 (9) | 6 |
| **Seven-category scales** |  |  |  |
| 3, No. (%) | 22 (19) | 19 (17) | 3 |
| 4, No. (%) | 79 (69) | 79 (69) | 0 |
| 5, No. (%) | 10 (9) | 12 (10) | 2 |
| 6, No. (%) | 3 (3) | 5 (4) | 2 |
| **Disease onset days** |  |  |  |
| < 7 days | 17 (15) | 20 (17) | 3 |
| 7-14 days | 97 (85) | 95 (83) | 3 |
| **Treatment, No. (%)** |  |  |  |
| Antiviral treatment | 95 (83) | 97 (84) | 1 |
| Arbidol | 66 (58) | 73 (63) | 6 |
| IFN-α | 36 (32) | 34 (30) | 1 |
| LPV/r | 35 (31) | 29 (25) | 5 |
| RBV | 9 (8) | 11 (10) | 2 |
| OSV | 33 (29) | 30 (26) | 3 |
| Antibiotics | 107 (94) | 100 (87) | 7 |
| Antifungal | 18 (16) | 13 (11) | 5 |
| TCM | 57 (50) | 56 (49) | 2 |
| LMWH | 18 (16) | 19 (17) | 1 |
| Glucocorticoids | 72 (63) | 73 (63) | 0 |

**Table S4 Clinical outcomes of all patients after the inverse probability of treatment weight–adjusted**

|  | **IVIg group** | **Control group** | **Absolute difference**  (95% CI) | ***P* value** |
| --- | --- | --- | --- | --- |
| **Primary outcome** |  |  |  |  |
| 28 days mortality, No. (%) | 3 (3%) | 31 (27%) | -25% (-34% to -16%) | <0.001 |
| **Secondary outcomes** |  |  |  |  |
| Time to clinical improvement, days (IQR) | 14 (12-22) | 18 (14-22) | -2 (-6 to 4) | 0.910 |
| Clinical improvement, No. (%) |  |  |  |  |
| Day 7 | 11 (10%) | 0 (0%) | 10% (4% to 15%) | 0.001 |
| Day 14 | 40 (35%) | 34 (30%) | 4% (-8% to 16%) | 0.537 |
| Day 21 | 70 (61%) | 55 (48%) | 14% (1% to 27%) | 0.034 |
| Day 28 | 97 (85%) | 67 (58%) | 26% (15% to 38%) | <0.001 |
| Duration of mechanical ventilation, days (IQR) | 13 (4-31) | 9 (4-10) | 4 (0 to 8) | 0.350 |
| Duration of hospitalization in survivors, days (IQR) | 18 (15-26) | 25 (17-28) | -2 (-4 to 1) | 0.143 |
| Duration of positive RT-PCR results, days (IQR) | 10 (7-16) | 10 (5-15) | 2 (-1 to 5) | 0.041 |
| Time to inflammatory markers normalization, days (IQR) |  |  |  |  |
| IL-6 | 4 (4-6) | 11 (7-18) | -6 (-8 to -3) | <0.001 |
| IL-8 | 5 (4-5) | 5 (2-11) | -3 (-5 to -1) | 0.001 |
| IL-10 | 5 (4-5) | 6 (4-11) | -4 (-6 to -2) | 0.001 |
| TNF-α | 5 (4-11) | 9 (4-16) | -3 (-7 to 1) | 0.092 |
| hsCRP | 11 (4-14) | 14 (9-18) | -3(-6 to 0) | 0.056 |
| Serum ferritin | 6 (5-6) | 12 (7-21) | -8 (-12 to -5) | <0.001 |
| ESR | 11 (11-13) | 14 (6-26) | -4 (-10 to 2) | 0.141 |

**Table S5. Multivariable cox regression analysis of inflammatory markers normalization of patients.**

| **Variables** | **Unadjusted model** | | **Demographic-adjusted model** | | **Fully-adjusted model** | |
| --- | --- | --- | --- | --- | --- | --- |
|  | HR (95.0% CI) | P value | HR (95.0% CI) | P value | HR (95.0% CI) | P value |
| **IL6** |  |  |  |  |  |  |
| Origin cohort | 3.11 (1.33-7.26) | 0.009 | 3.13 (1.34-7.33) | 0.009 | 3.79 (1.10-12.08) | 0.035 |
| IPTW model | 3.49 (2.04-5.97) | <0.001 | 3.71 (2.15-6.40) | <0.001 | 3.85 (2.68-12.78) | <0.001 |
| IPTW-MI model | 2.44 (1.19-4.99) | 0.015 | 2.60 (1.29-5.24) | 0.007 | 2.88 (1.08-7.99) | 0.047 |
| **IL10** |  |  |  |  |  |  |
| Origin cohort | 2.21 (1.14-4.29) | 0.019 | 2.18 (1.12-4.23) | 0.022 | 1.43 (0.50-4.10) | 0.509 |
| IPTW model | 2.14 (1.42-3.21) | <0.001 | 2.27 (1.49-3.46) | <0.001 | 1.76 (0.89-3.53) | 0.111 |
| IPTW-MI model | 1.99 (1.18-3.27) | 0.009 | 1.72 (0.98-2.99) | 0.057 | 1.49 (0.25-1.77) | 0.418 |
| **Ferritin** |  |  |  |  |  |  |
| Origin cohort | 4.33 (1.38-8.02) | 0.007 | 4.42 (1.40-8.33) | 0.007 | 7.21 (1.45-35.74) | 0.016 |
| IPTW model | 4.76 (3.35-9.91) | <0.001 | 4.76 (3.35-9.91) | <0.001 | 6.77 (3.41-13.48) | <0.001 |
| IPTW-MI model | 6.12 (2.91-12.86) | <0.001 | 18.06(6.36-51.23) | <0.001 | 7.71 (2.11-14.15) | 0.068 |

Demographic-adjusted: controlled for age and sex as covariates; Fully-adjusted: controlled for age, sex, comorbidity, disease onset days, baseline seven scale category, the use of arbidol, LPV/r, IFN, RBV, OSV, antibiotics, antifungals, TCM, glucocorticoids, LMWH as covariates.

**Figure S1. Distributions of propensity scores between the two treatment groups.**


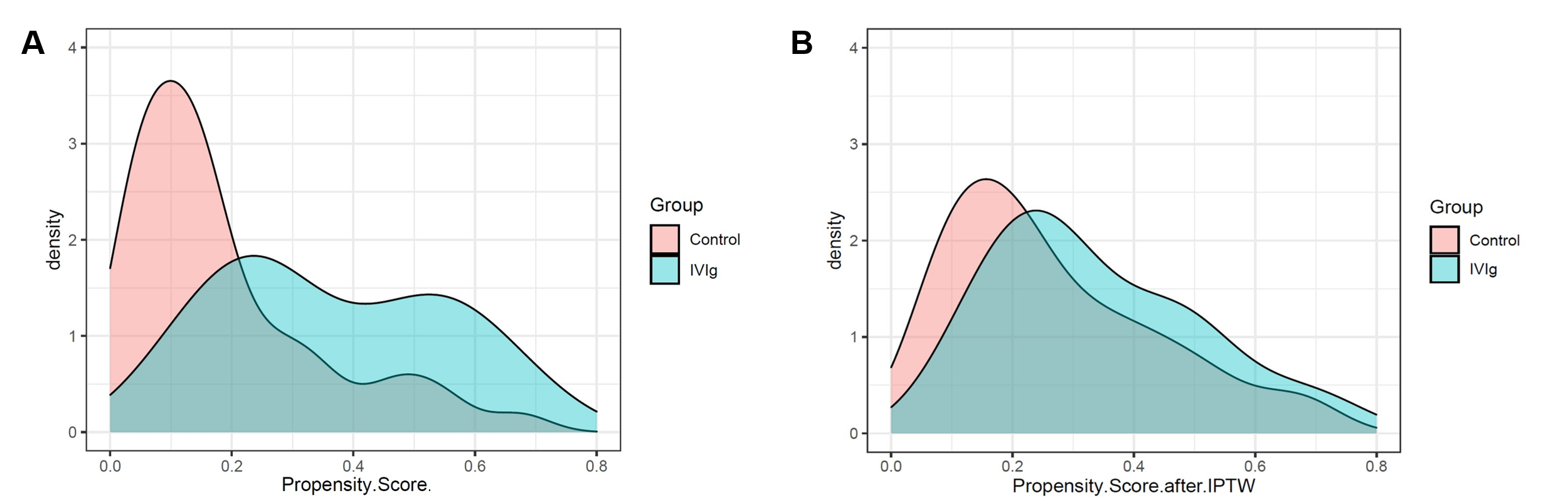
 The distributions of both groups were similar according to the propensity score after IPTW.

**Figure S2. Monitoring of laboratory examination among IVIg treated patients.**

**Figure S3. Monitoring of laboratory examination among IVIg treated patients.**
